# Tuina for diabetic peripheral neuropathy: A protocol for a systematic review and meta-analysis

**DOI:** 10.1097/MD.0000000000026222

**Published:** 2021-06-11

**Authors:** Fengyang Wang, Fengjuan Wang, Ting Pan, Zhenzhong Wu, Yufeng Wang, Peng Liu, Ziyang Yu, Rui Shang, Bailin Song

**Affiliations:** a Department of Acupuncture and Tuina, Changchun University of Chinese Medicine; b Anesthesia professional, Traditional Chinese Medicine Hospital of Jilin Province; c Anesthesia professional, Jilin Qianwei Hospital; d Department of Tuina, Traditional Chinese Medicine Hospital of Jilin Province; e Endocrinology of Traditional Chinese Medicine, Changchun University of Chinese Medicine; f Department of Acupuncture and Tuina, Jilin Provincial Cancer Hospital; g Traditional Chinese Medicine, Changchun University of Chinese Medicine, Changchun, China.

**Keywords:** diabetic peripheral neuropathy, effectiveness, protocol, safety, systematic review, tuina

## Abstract

**Background:**

Diabetic peripheral neuropathy (DPN) is one of the most common microvascular complications of diabetes mellitus, with an incidence ranging from 60% to 90%. With the change in modern dietary structure, the incidence of diabetes is increasing year by year, and DPN is also on the rise. Tuina therapy has been widely used in the treatment of DPN, but there is no systematic review on the treatment of DPN. Therefore, this study aimed to conduct a meta-analysis of Tuina in the treatment of DPN to clarify its efficacy.

**Methods:**

The following electronic databases will be searched: PubMed, the Cochrane Library, Embase, Web of Science, Medline, CNKI, Chinese Biomedical Literature Database, VIP, and Wan Fang databases. We will consider articles published between database initiation and May 2021. We will use Review Manager 5.4, provided by the Cochrane Collaborative Network for statistical analysis. Clinical randomized controlled trials related to Tuina for diabetic peripheral neuropathy were included in this study. Language is limited to both Chinese and English. Research selection, data extraction, and research quality assessments were independently completed by two researchers. We then assessed the quality and risk of the included studies and observed the outcome measures.

**Results:**

This study provides a high-quality synthesis to assess the effectiveness and safety of Tuina for treating diabetic peripheral neuropathy.

**Conclusion:**

This systematic review will provide evidence to determine whether Tuina is an effective and safe intervention for patients with diabetic peripheral neuropathy.

**Ethics and dissemination:**

The protocol of the systematic review does not require ethical approval because it does not involve humans. This article will be published in peer-reviewed journals and presented at relevant conferences.

**Registration number:**

INPLASY202150027.

## 1 Introduction

Diabetic peripheral neuropathy (DPN) is a common chronic complication of diabetes mellitus. The incidence of DPN has been reported to be >50%.^[1–3]^ Diabetic peripheral neuropathy (DPN) is a major cause of reduced quality of life due to pain, sensory loss, gait instability, fall-related injury, foot ulceration, and amputation.^[4–5]^ Early peripheral neuropathy may present as sensory alterations that are often progressive, including sensory loss, numbness, pain, or burning sensations in a “stocking and glove” distribution of the extremities. Later stages may involve proximal numbness, distal weakness, or atrophy. Physical examination should include comprehensive neurologic and musculoskeletal evaluations.^[6–9]^

Studies have shown that poor glycemic control is a risk factor for DPN, but other risk factors are also involved.^[10]^ Recently, other studies have also implicated cardiovascular risk factors, such as obesity and triglycerides.^[11–12]^ There is also evidence that DPN is associated with cardiovascular disease and mortality.^[13]^ DPN treatment mainly includes the cause of treatment, symptomatic treatment, and strict control of hyperglycemia and other controllable neuropathy risk factors, nutritional nerve, antioxidant stress, improving microcirculation, etc. the treatment effect is not good.^[14–17]^ Massage can effectively relieve the symptoms of diabetic peripheral neuropathy.^[18]^ Therefore, the use of Tuina in DPN therapy requires further research. At present, there is no systematic review of Tuina treatment for the treatment of diabetic peripheral neuropathy, so this study will evaluate the efficacy and safety of Tuina in the treatment of diabetic peripheral neuropathy, and provide evidence for clinical decision-making in Tuina.

## 2 Methods and analysis

The systematic review will be performed following the guidelines of the preferred reporting items for systematic review and meta-analysis protocols (PRISMA-P) 2015.^[19]^ This protocol was registered on the international platform of registered systematic review and meta-analysis protocols (INPLASY202150027).

### 2.1 Inclusion criteria

#### 2.1.1 Types of participants

Studies on adult patients diagnosed with DPN are included in this study. No limitations of location, educational background, and gender were imposed.

#### 2.1.2 Types of interventions

The treatment group using Tuina, while the control group received treatment with oral medication, acupuncture, Chinese herbal medication, physical therapy, Botox injections, and so on, or even with no treatment, will be included.

#### 2.1.3 Types of studies

This review will include randomized controlled trials (RCTs) on Tuina for DPN published in Chinese and English, respectively. We will exclude non-RCTs, review studies, case reports, and animal experiments.

#### 2.1.4 Types of outcomes

The primary outcome included the glycemic profile, as measured by fasting blood glucose or glycated hemoglobin. The secondary outcomes consisted of neuropathic pain intensity, as assessed by visual analog scale or other relevant tools; plantar tactile sensitivity, as evaluated by Semmes-Weinstein monofilament; sensory nerve conduction velocity and motor nerve conduction velocity, as assessed by electromyography; quality of life, as evaluated by health-related quality of life scale or associated scores, and adverse events.

### 2.2 Data sources and search methods

#### 2.2.1 Electronic searches

This study will use the PubMed, Cochrane Library, Embase, Web of Science, and Medline databases. In addition, we will also collect four databases of China: China National Knowledge Infrastructure, China Biomedical Literature Database, VIP Database, and Wan-fang Database. We will consider articles published between database initiation and May 2021. The search terms were diabetic neuropathy, peripheral neuropathy, neuropathy, diabetic neuropathy, diabetic neuropathy, diabetic neuropathy, diabetes mellitus, Tuina, Tuina therapy, etc. The search strategy for PubMed is presented in Table 1. Similar research strategies for other electronic databases are adapted and applied.

**Table 1:** Search strategy for the PubMed database.

Number	Terms
#1	diabetic neuropathy (all field)
#2	peripheral neuropathy (all field)
#3	diabetic polyneuropathy (all field)
#4	diabetic neuropathies (all field)
#5	diabetes mellitus (all field)
#6	diabetic (all field)
#7	neuropathy (all field)
#8	#1 OR #2–7
#9	Tuina (all field)
#10	massage (all field)
#11	massotherapy (all field)
#12	knead (all field)
#13	rub (all field)
#14	massieren (all field)
#15	massaging (all field)
#16	Manipulation (all field)
#17	#9 OR #10–16
#18	randomized controlled trial (all field)
#19	randomly (all field)
#20	controlled clinical trial (all field)
#21	randomized (all field)
#22	random allocation (all field)
#23	placebo (all field)
#24	single-blind method (all field)
#25	double-blind method (all field)
#26	trials (all field)
#27	comparators
#28	allocation
#29	#18 OR #19–28
#30	#8 And #17 And #29

#### 2.2.2 Searching for other resources

We will search for a list of related references for additional trials. The PubMed and Cochrane Library will be searched for existing systematic reviews related to our topic to search their reference lists for further studies. We will also search a reference list for identifying published journals, books, conference articles, and gray literature related to this research topic.

### 2.3 Data selection

**Figure 1:**
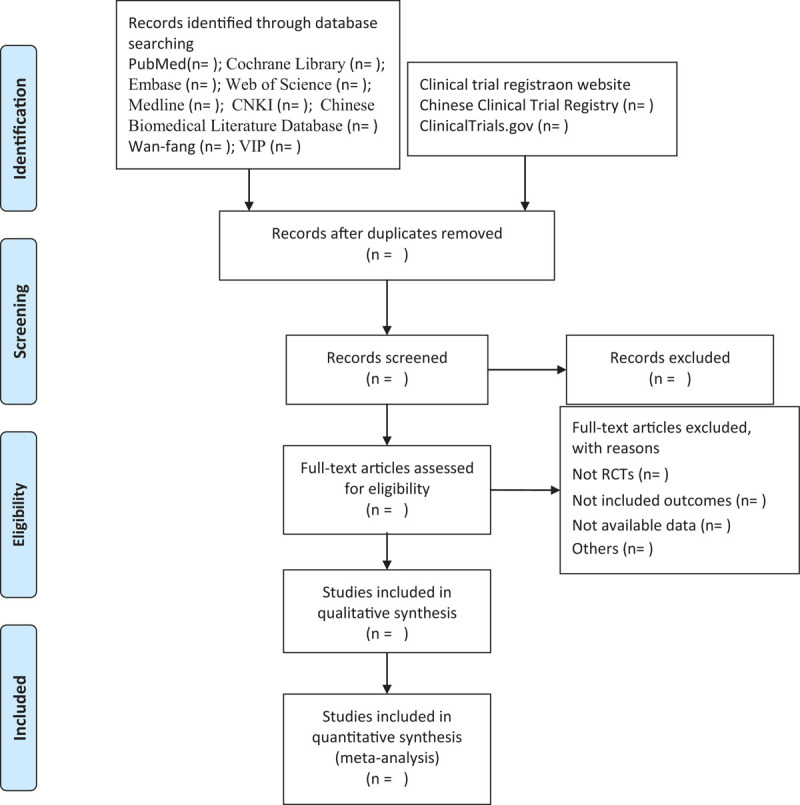
Flow diagram of the study selection process.

Researchers will import all the literature into Endnote X9 software for collation, and repetitive studies will be deleted by the software. First, we conduct a preliminary screening of the literature. The researchers screened the literature that met the inclusion criteria by reading the title and abstract. Finally, we will conduct a depth screening for the preliminary screening of the literature that meets the criteria, and browse the full text more thoughtfully and carefully to further determine whether to include or exclude. Finally, the final included literature will be exchanged and checked by researchers. If the two researchers disagree on the results of a study or eventual inclusion, we will resolve it through discussion or consultation with a third person. A flowchart of the screening process of the study is shown in Figure 1.

### 2.4 Data extraction and analysis

Before data collection, our study team built a data extraction sheet. Two authors separately collected relevant information from each eligible study. The following information was extracted: journal-title, first author, year of publication, study design, patient characteristics, control intervention, experimental intervention, outcomes, and duration of intervention. If a study has unclear or inadequate information, we will attempt to contact the authors via email. Any dispute was resolved by consulting a third reviewer.

### 2.5 Risk of bias assessment

Two reviewers will separately assess the risk of bias of the selected RCTs using the Cochrane risk of the bias assessment tool. The evaluation of each study mainly included the following seven aspects: random sequence generation, allocation hiding, blinding of participants and personnel, blinding of outcome assessment, incomplete outcome data, incomplete outcome data, selective outcome reporting, and other biases. A bias value of “low” “unclear” or “high” will be used to rank the risk of bias. These even domains will be separately appraised by 2 reviews, and discrepancies will be addressed by consulting a third reviewer.

### 2.6 Data synthesis and analysis

ReMan 5.4 software will be used for data synthesis and meta-analysis. The heterogeneity of data was tested by calculating the I^2^ statistic. The study was not considered to have a large heterogeneity when the I^2^ value was less than 50%. when the I^2^ value exceeded 50%, there was significant statistical heterogeneity among the trials. When there is homogeneity in the merged outcome results across sufficient studies, a meta-analysis will be conducted. Otherwise, we will carry out a subgroup analysis to explore the causes of obvious heterogeneity.

### 2.7 Assessment of reporting biases

We used funnel charts to assess reporting biases. When a sufficient number of included studies (at least 10 trials) are available, we will conduct a test for funnel plot asymmetry using the Egger method.

### 2.8 Subgroup analysis

If necessary, subgroup analysis will be conducted based on the different study qualities, interventions, controls, and outcome measurements.

### 2.9 Sensitivity analysis

We conducted a sensitivity analysis according to the recommendations of the Cochrane Handbook to evaluate the quality and robustness of the merger results of the whole study. The main analysis points included the impact of method quality, sample size, and missing data on the study. The meta-analysis will be reused, and more inferior-quality studies will be excluded. The results are compared and discussed based on the results.

### 2.10 Grading the quality of evidence

The quality of systematic reviews will be evaluated using the grading of recommendations, assessment, development, and evaluation. It is evaluated according to the five aspects of the study: limitations, inconsistencies, indirectness, inaccuracy, and publication bias of the research design. In the end, the quality of the research will be divided into 4 levels from high to low are high, medium, low, and very low.

### 2.11 Ethics and dissemination

This meta-analysis was not required for ethical approval of the published data. Our findings will be published in peer-reviewed journals.

## 3 Discussion

Diabetic peripheral neuropathy is a common complication in patients with diabetes, which increases the difficulty of clinical treatment.^[20]^ Diabetic peripheral neuropathic pain affects the functionality, mood, and sleep patterns of approximately 10% to 20%of patients with diabetes mellitus.^[21]^ Hyperglycemia plays a key role in the development and progression of diabetic neuropathy.^[22]^ Currently, medication management is widely used for this condition; however, there are still some shortcomings, such as limited effectiveness and severe side effects.^[23]^ Tuina is a traditional Chinese physical therapy, which can improve the curative effect, shorten the course of treatment, are simple to operate, have few side effects, and have a significant effect on the treatment of DPN. Previous studies have reported that Tuina can benefit patients with DPN. However, no systematic review has explored this issue. Thus, this study is the first to systematically investigate the effectiveness and safety of Tuina for the treatment of patients with DPN. The results of this study will provide helpful evidence for both clinical practice and future studies.

## Author contributions

**Data curation:** Yufeng Wang, Ziyang Yu.

**Formal analysis:** Ting Pan, Rui Shang.

**Funding acquisition:** Bailin Song.

**Investigation:** Fengyang Wang, Peng Liu.

**Methodology:** Fengjuan Wang, Zhenzhong Wu.

**Validation:** Yufeng Wang.

**Writing – original draft:** Fengyang Wang.

**Writing – review & editing:** Yufeng Wang.
